# Single-Step Fabrication of Polymer Nanocomposite Films

**DOI:** 10.3390/ma11071177

**Published:** 2018-07-10

**Authors:** Christoph O. Blattmann, Sotiris E. Pratsinis

**Affiliations:** Particle Technology Laboratory ETH Zürich, Sonneggstrasse 3, 8092 Zürich, Switzerland; blattmann@ptl.mavt.ethz.ch

**Keywords:** nanoparticles, patterned composites, additive manufacturing, homogeneous, multifunctional

## Abstract

Polymer nanocomposites are employed in (micro)electronic, biomedical, structural and optical applications. Their fabrication is challenging due to nanoparticle (filler) agglomeration and settling, increased viscosity of blended solutions and multiple tedious processing steps, just to name a few. Often this leads to an upper limit for filler content, requirements for filler–polymer interfacial chemistry and expensive manufacturing. As a result, novel but simple processes for nanocomposite manufacture that overcome such hurdles are needed. Here, a truly single-step procedure for synthesis of polymer nanocomposite films, structures and patterns at high loadings of nanoparticles (for example, >24 vol %) for a variety of compositions is presented. It is highly versatile with respect to rapid preparation of films possessing multiple layers and filler content gradients even on untreated challenging substrates (paper, glass, polymers). Such composites containing homogeneously dispersed nanoparticles even at high loadings can improve the mechanical strength of hydrogels, load-bearing ability of fragile microstructures, gas permeability in thin barriers, performance of dielectrics and device integration in stretchable electronics.

## 1. Introduction

Preparation of polymer nanocomposite films or coatings has been an ongoing challenge so that no optimal fabrication method has yet evolved. All of them generally aim for well-dispersed nanoparticles (filler) within the polymer regardless of filler loading [1]. However, especially at higher loadings, where the sought effect of the nanoparticles becomes stronger [2], their agglomeration increases, diminishing the composite applicability [3]. Typically, this requires chemically modifying the nanoparticle surface so that more homogeneous dispersion within that polymer [1] is obtained, leading to frequent development of tedious and expensive fabrication methods.

Once overcoming the hurdle of agglomeration, blending filler with polymer encounters rising composite viscosity with increasing nanoparticle content [4]. Then, more energy-intensive mixing equipment and longer durations become unavoidable [5]. Preparation of thin films from such viscous solutions, for example, by spin-coating [6], is difficult. Addition of a solvent (i.e., polymer–nanoparticle ink) can reduce this rise in viscosity [7]. However, quick settling or agglomeration of the nanoparticles, for example, during lengthy processing, solvent evaporation or storage, again may incur inhomogeneity within the composite. Furthermore, agglomerated nanoparticles in such inks can clog dispensing equipment [8].

In-situ formation of nanoparticles in a polymer matrix [9] or vapor co-deposition of the two components [10] are both techniques for which agglomerate-free composites at high filler loadings are feasible. These techniques, however, are applicable mostly for spherical metallic nanofillers whose dimensions increase with their content. The former technique [9] suffers additionally from chemical residues remaining after nanoparticle formation. When employing the latter [10], on the other hand, the property gain must be in balance with their lengthy fabrication and expensive vacuum equipment.

**Figure 1:**
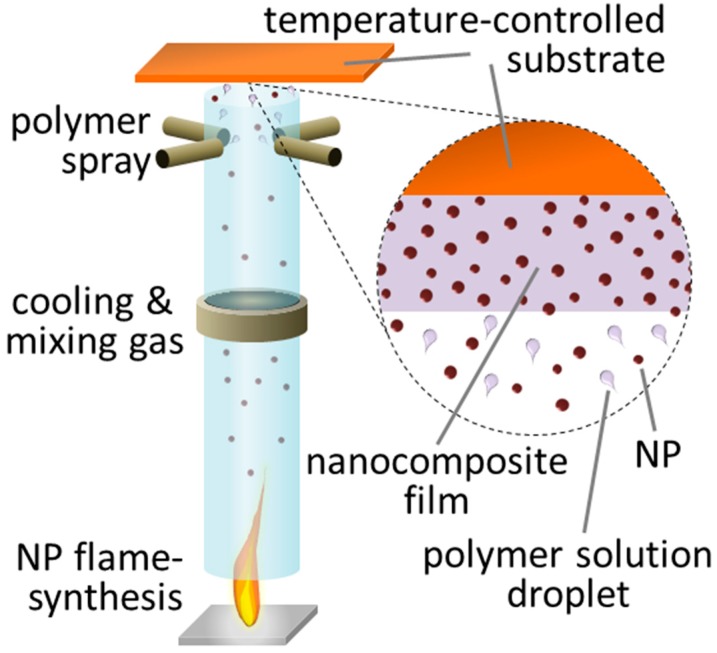
Set-up for of single-step nanocomposite fabrication combining flame synthesis of nanoparticles (filler) with simultaneous spraying of a polymer solution. Freshly-made nanoparticles (NPs) and polymer deposit on a temperature-controlled substrate forming a nanocomposite film.

A long-standing challenge for the fabrication of polymer nanocomposite films is the development of a scalable manufacturing technique which can be used directly for a wide variety of polymers and filler (size, material, composition) at high loading without agglomeration. The present work introduces a truly single-step technique for rapid fabrication of such polymer nanocomposite films. The technique combines flame synthesis of nanoparticles by flame-spray pyrolysis [11] with simultaneous spraying of a polymer solution (Figure 1) similar to that for continuous surface functionalization of titania [12] or preparation of magnetic epoxy [13]. More specifically, freshly made aerosol nanoparticles are cooled and mixed [12] in-flight by an upward draft through guiding tubes created from a vortex swirl nozzle [14] injecting a high flow-rate of gas. Downstream, a dilute polymer solution (aqueous in this work) is sprayed through four symmetrically aligned dispersion nozzles and mixed with the aerosol nanoparticles. Vertically above the tube exit, this aerosol mixture impinges onto a temperature-controlled substrate [15] maintained, say, at 110 °C. There, the polymer-carrying solution evaporates. As a result, a polymer nanocomposite film gradually forms on the desired substrate. Its composition equals that of the freshly flame-synthesized nanoparticles and the unmodified commercial polymer. Here, the substrate size is about 9 cm^2^ but larger areas can be made with a motorized substrate suspension [16]. The technique can be extended to additionally incorporate in-situ-formed nanoparticles by modifying the polymer solution [9].

In contrast to the majority of polymer nanocomposite fabrication methods, the one described here is truly single-step since nanoparticle synthesis, mixing with polymer and composite deposition are conducted simultaneously. This is beneficial since greater material losses, contamination and labor time by multiple fabrication steps increase chances of processing failure and the amount of required infrastructure and chemicals that skyrocket the product cost. These considerations become important when assessing the potential for large-scale fabrication.

## 2. Materials and Methods

### 2.1. Materials

Poly(vinyl alcohol) (PVA; PolySciences; MW = 133,000 g·mol^−1^; 99% hydrolyzed) and iron(III) acetylacetonate (>99%) were purchased from Chemie Brunschwig AG (Basel, Switzerland) while O_2_, N_2_, CH_4_ were purchased from PanGas AG (Muttenz, Switzerland). Pressure air from household internal supply was used. Ethanol (A15-A, 98%) was purchased from AlcoSuisse AG (Bern, Switzerland). All other chemicals were purchased from Sigma-Aldrich Chemie GmbH (St. Louis, MO, USA).

### 2.2. Fabrication

Unless otherwise stated, SiO_2_ and TiO_2_ precursors consist of hexamethyldisiloxane (>98%) (Si concentration C_f_ = 0.25 M) and titanium(IV) isopropoxide (>97%) (Ti concentration C_f_ = 0.25 M), respectively, dissolved in ethanol and xylene (>98.5%) (1:1 vol. ratio). The γ-Fe_2_O_3_ precursor solution is prepared with 0.3 M iron(III) acetylacetonate in mixture of acetonitrile (>99.9%), ethanol and xylene (1:1:2 vol. ratio). A standard flame-spray pyrolysis burner [17] is used for nanoparticle synthesis. It is operated with 5 mL·min^−1^ precursor and 5 L·min^−1^ O_2_ dispersion flow (1.5 bar back-pressure). Ignition of this spray occurs by a methane pilot flame (1.5 L·min^−1^ CH_4_ and 3.2 L·min^−1^ O_2_).

A 30-cm-long quartz tube (50 mm diameter) is centered vertically above the flame with a lift-off distance [18] of 3 cm. A 16-hole vortex-swirl nozzle [14] fed with 65 L·min^−1^ pressure air (20 °C) and a 20-cm-long steel tube (50 mm diameter) are fastened in this sequence directly to the exit of the quartz tube. During nanocomposite deposition the substrate (usually 1 mm-thick borosilicate glass cleaned by sonication in ethanol) is centered above the steel tube exit with 1.6 cm separation distance. It is fastened to a copper block kept at 110 °C by integrated thermocouple-controlled heating and water cooling [15].

The PVA or poly(3,4-ethylenedioxythiophene):poly(styrenesulfonate) (PEDOT:PSS; high-conductivity grade, Orgacon^TM^ HIL-1005) solutions are sprayed radially into the tube system 4 cm below the steel tube exit through four symmetrically positioned dispersion nozzles. Each nozzle is fed with 2 mL·min^−1^ of dilute polymer solution and dispersed by 5 L·min^−1^ N_2_ (1 bar back-pressure). Polymers are dissolved in a water and ethylene glycol (>99.5%) mixture (7:1 vol. ratio). The polymer solution concentration (C_p_) of the PVA solution ranges from 0 to 1/8 wt % while 1/16 wt % is used for the PEDOT:PSS solution.

### 2.3. Characterization

Imaging by field emission scanning electron microscopy (FE SEM) was done with a Hitachi S-4800 FE SEM (Hitachi Ltd., Tokyo, Japan). Measurements from these images (for example, nanocomposite film thickness) were conducted with ImageJ software (v. 1.42q). Filler content was measured by thermogravimetric analysis in air (NETZSCH STA 449 C, NETZCH-Gerätebau GmbH, Selb, Germany). Particle size (d_SiO2_) was determined by Brunauer-Emmett-Teller (BET)-N_2_ adsorption (Micromeritics TriStar, Micromeritics, Norcross, GA USA) from filter-collected polymer-free (C_p_ = 0 wt %) SiO_2_ nanoparticles [11]. Resistance measurements were done with a Tektronix DMM4050 multimeter (Tektronix Inc., Beaverton, OR, USA).

## 3. Results and Discussion

### 3.1. Films with Tunable High Loading of Filler Nanoparticles

**Figure 2:**
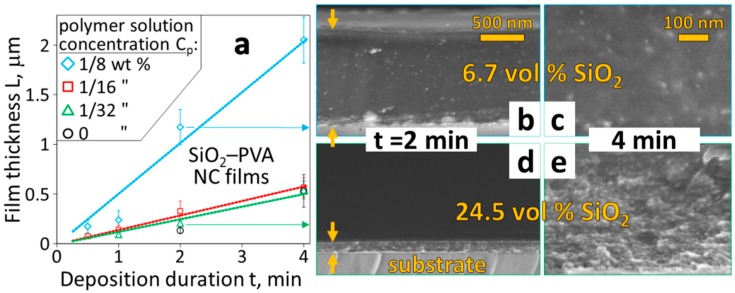
(**a**) The SiO_2_ nanocomposite (NC) film thickness L as a function of deposition duration t for varying PVA concentrations C_p_ in polymer solutions (diamonds, squares, triangles, circles for C_p_ = 1/8, 1/16, 1/32 and 0 wt %, respectively). (**b**–**e**) SEM cross-section images of these films deposited on glass for t = 2 (**b**,**d**) and 4 min (**c**,**e**) with C_p_ = 1/8 (**b**,**c**) and 1/32 wt % (**d**,**e**). Smooth surface and homogeneous SiO_2_ nanoparticle distribution (bright dots in images) within the PVA (dark grey surrounding matrix) are seen in these void-free films. Scale bars in (**d**,**e**) same as in (**b**,**c**), respectively.

The fabrication setup (Figure 1) combines independently adjustable nanoparticle synthesis and polymer solution spraying. This enables free selection of the size, morphology and composition of nanoparticles, their generation rate as well as the polymer matrix and final filler content. Figure 2a shows the film thickness L as a function of deposition duration t for a nanocomposite made of polyvinyl alcohol (PVA) and silica (SiO_2_) nanoparticles. The filler loading is varied by employing different solutions of low polymer (PVA) concentrations C_p_ = 0, 1/32, 1/16 and 1/8 wt %. The L increases with t independent of C_p_ so that nanocomposite thicknesses of tens to thousands of nanometers are realized.

These films are homogeneously thick with a smooth surface as can be seen in the cross-section images of Figure 2b,d (t = 2 min for C_p_ = 1/8 and 1/32 wt %, respectively). Their visually determined high transparency (especially at high C_p_) indicates that no entrapped air or voids are formed. At high C_p_, e.g., 1/8 wt % (diamonds), the film thickness L increases more rapidly (as expected) than at low C_p_ due to higher polymer feed-rate. In contrast, though, L increases only marginally faster for C_p_ = 1/16 wt % (squares) than the more dilute PVA solution (C_p_ = 1/32 wt %, (triangles). In fact, this rate is even quite similar to that when adding far less (C_p_ = 1/128 wt %, Figure S1) or no PVA at all (circles and Figure S2). Correspondingly, one can infer high SiO_2_ filler loading at these low C_p_ values (Figure S3).

The high filler loading in these films is optically supported by a magnified cross-section image (Figure 2e, C_p_ = 1/32 wt % and t = 4 min). The closely packed SiO_2_ (bright dots) within the PVA matrix (surrounding dark grey) can be identified. This image strongly contrasts to the equivalent film with much lower filler loading prepared with a quadrupled C_p_ (Figure 2c, t = 4 min). The nanoparticle distribution within the PVA, on the other hand, remains homogeneous regardless of C_p_.

Thermogravimetric analysis of multiple nanocomposite films prepared with C_p_ = 1/8 and 1/32 wt % revealed predictable (Figure S3) filler loading of 6.7 and 24.5 vol %, respectively. The former loading can be obtained by existing techniques [4] with limited agglomeration. The 24.5 vol %, however, is increasingly difficult to achieve [4] with such small fillers (d_SiO2_ = 20 nm) at this high homogeneity. These thermogravimetrically verified filler contents, however, are lower than expected from the mass balance, which indicates inefficient nanocomposite fabrication due to not-yet optimized synthesis conditions.

The ability to rapidly prepare homogeneous and highly loaded nanocomposites is quite helpful for realizing electrically conductive films [6], cantilevers [19], gas barriers [20], dielectric elastomers [21] and shape memory polymers [3] because agglomerating nanoparticles, formation of voids and/or difficulties during fabrication have limited the filler content [22]. Small nanoparticles (<20 nm) are especially difficult to incorporate homogeneously above 7 vol % into a polymer due to their high specific surface area [4]. High filler loading is possible by infiltration of a polymer solution into a dense percolating particle layer [2] but it is plagued by void/bubble formation and consists of multiple separate fabrication steps [23].

### 3.2. Multilayered Filler Content Gradients and Microstructures

**Figure 3:**
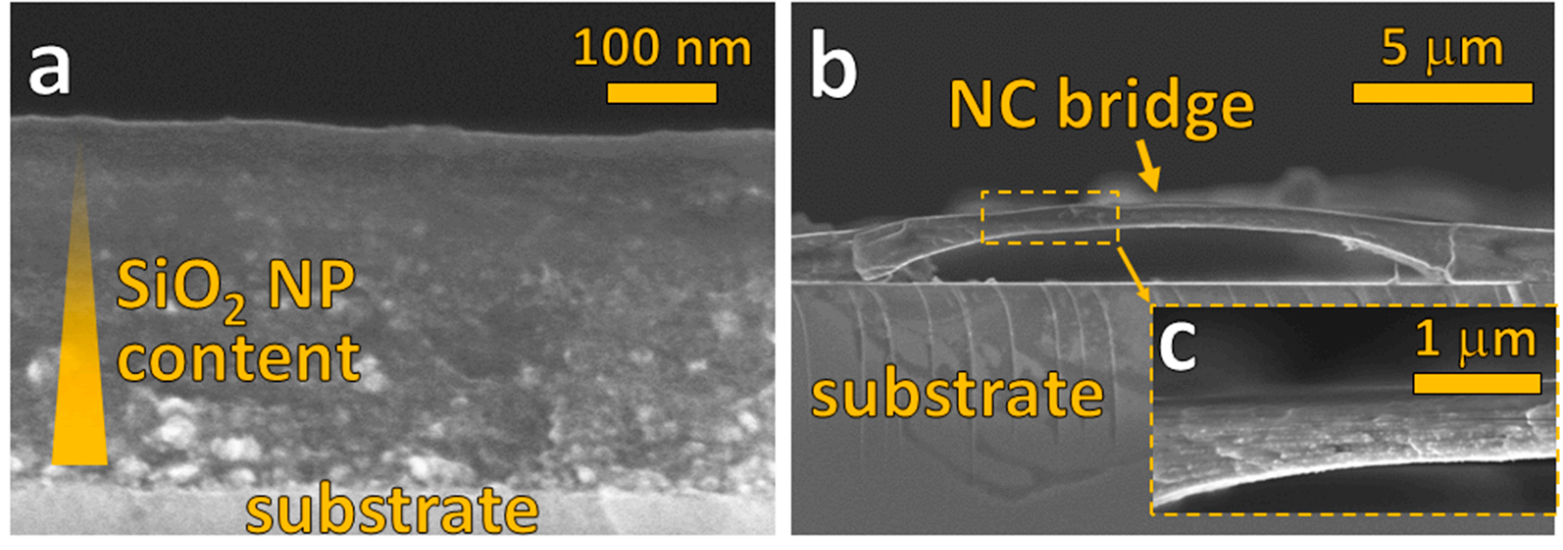
(**a**) SEM cross-section image of a SiO_2_–PVA nanocomposite film with filler content gradient (decreasing from substrate up). This was obtained by depositing four consecutive layers with progressively lower SiO_2_ content (t = 1 min each; C_p_ = 1/32 wt %) by reducing the concentration of Si precursor (C_f_ = 0.5 to 0.06 M) fed to the flame. (**b**) SiO_2_–PVA nanocomposite bridge of about 15 μm length prepared by deposition (t = 3 min; C_p_ = 1/8 wt %) on a patterned substrate. The homogeneous nanoparticle distribution of this submicrometer-thin bridge is seen in the enlarged area (**c**).

Sophisticated or optimized devices incorporating polymer nanocomposites cannot rely merely on a simple film with high spatial filler homogeneity. More preferable, for example, are vertical filler content gradients that increase or decrease gradually the mechanical Young’s modulus or yield [24] or improve breakdown strength and electrical insulation in dielectric nanocomposites [25]. Here, a filler content gradient in a single film is realized by subsequent deposition of multiple layers of nanocomposites creating a gradual decrease of filler content. Figure 3a shows a cross-section image of this nanocomposite consisting of four SiO_2_ nanoparticle–PVA layers. The filler content is decreased for each subsequently deposited layer (i.e., from substrate up) by reducing the concentration of the Si precursor (C_f_ = 0.5, 0.25, 0.13, 0.06 M) fed to the flame. These layers blend well into each other so that one cannot recognize the transition of filler content at individual layer interfaces by SEM. The diminishing SiO_2_ content, on the other hand, is observed distinctly in subsequently deposited layers from the smoother cross-section interface and fewer bright spots (corresponding to SiO_2_ nanoparticles).

It must be stressed that this multilayer fabrication does not require a time gap between each layer for drying [25] or curing of the nanocomposite. Also, the nanoparticle precursor and/or polymer solution can be readily altered during deposition. This reduces the overall processing time that is especially important for large scale manufacture.

Fabrication of nanocomposites for micro-electronics or -fluidics demands compatibility with patterning and etching to create intricate structures at the micron scale [26]. Here a SiO_2_–PVA nanocomposite bridge of several micrometers in length and less than 1 µm in thickness (Figure 3b,c) demonstrates such compatible fabrication. This bridge was created by patterning a clean glass substrate with poly(methyl methacrylate) (PMMA) through a polyimide mask, depositing the nanocomposite on top and finally removing the PMMA in an acetone bath. The homogeneously distributed silica nanoparticles most likely contribute to increased mechanical strength [27] of the bridge (not determined in this work). More precise and functional structures may be made with a lithographically prepared substrate.

### 3.3. Free-Standing and PDMS-Embedded Nanocomposites

**Figure 4:**
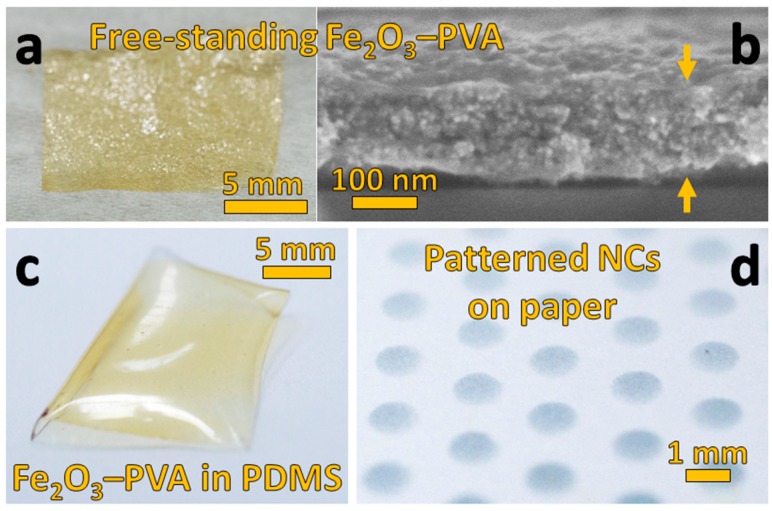
(**a**) Free-standing γ-Fe_2_O_3_–PVA film (t = 3 min; C_p_ = 1/32 wt %) after removal from the sacrificial layer-coated substrate (now on Kimtech^®^ tissue). The cross-section of this film (**b**) depicts the high homogeneity, large filler fraction and its <200 nm thickness. This same film was also prepared directly onto untreated PDMS. After additionally coating it with a transparent PDMS layer, this mechanically robust nanocomposite (**c**) is made free-standing. (**d**) Standard white printer paper (80 g·m^−2^) was used as substrate for circular TiO_2_–PEDOT:PSS nanocomposite (NC) array deposition (t = 1 min) through a shadow mask.

Detaching entire nanocomposite films from their fabrication substrate is essential when implantability, flexibility, light weight or high optical transparency are required. Figure 4a shows such a detached film deposited (Figure 1) on top of a sacrificial layer-coated substrate. This film not only has large area (~4 cm^2^), but also exhibits a thickness of less than 200 nm. Figure 4b shows a cross-section image of that film from which its slender dimension can be measured. Such thin and highly loaded nanocomposites are extremely difficult to prepare by coating techniques (for example, doctor blading, spin-coating) due to the rise in solution viscosity especially at high filler contents [6].

This film (Figure 4a,b) demonstrates an additional key benefit of the fabrication method (Figure 1): The range of filler characteristics is quite unlimited due to the myriad compositions, sizes and morphologies that can be made by flame aerosol technology [28]. For example, superparamagnetic iron oxide (γ-Fe_2_O_3_) nanoparticles [29] were incorporated into the PVA matrix which renders the yellow–brown color in Figure 4a. Their superior contrast compared to SiO_2_ (Figure 2 and Figure 3) makes them even more clearly discernible by SEM (Figure 4b). One can therefore more easily see that high filler loading without recognizable agglomeration is achieved.

Thin nanocomposite films at high filler content (Figure 4a,b) are mechanically fragile [30]. As a result, they are embedded frequently within a more durable matrix. Silicone rubber (PDMS) is widely used for this purpose for its mechanical stability and flexibility, high optical transparency and biocompatibility. The preparation of polymer nanocomposites on non-treated PDMS, on the other hand, is quite challenging [31] for solution-based techniques (for example, spin-coating, printing, spraying) due to its hydrophobicity and rapid swelling upon exposure to organic solvents. To show how this difficulty can be overcome, the bare γ-Fe_2_O_3_–PVA nanocomposite (Figure 4a) was prepared directly on PDMS (Figure 4c) without surface pretreatment. No loss of homogeneity occurs according to the even yellow–brown color of the film that is identical to that not prepared on PDMS (Figure 4a).

The simultaneous deposition of flame-made nanoparticles and polymer solution onto a temperature-controlled substrate (Figure 1) is key to enabling nanocomposite preparation on substrates like PDMS: Deposited nanoparticles lend PDMS a good surface wettability so that the polymer solution evenly spreads over the entire area in contrast to island formation [32]. This, however, only briefly is required due to ongoing drying of the polymer solution stimulated by the heated substrate. In the absence of nanoparticles, the surface wetting of the substrate is significantly reduced so that rather uneven film thicknesses are obtained even on bare glass (Figure S4). Adding only a small amount of nanoparticles eliminates this difficulty as indicated by the planar surface of the SiO_2_–PVA nanocomposite (Figure 3a) having a very low filler content in its top layer.

### 3.4. Patterned Nanocomposites on Paper

Like PDMS, paper is another attractive substrate [33] especially for nanocomposites in inexpensive and disposable devices or for labelling and authentication of packaging. In fact, directly depositing flame-synthesized TiO_2_ nanoparticles without a polymer already has been used to realize hydrophobic coatings on paper [34]. Figure 4d shows a photograph of an array of circular nanocomposites deposited on standard white printer paper (cellulose, 80 g·m^−2^). Their fabrication requires no change of parameters irrespective of the rough topography and high porosity of the fibrous paper substrate. These nanocomposites were prepared with a dilute PEDOT:PSS solution as polymer which leads to their blue color. This not only demonstrates the successful use of PEDOT:PSS, an attractive material for flexible electronics [31], but also shows that this single-step nanocomposite fabrication method is compatible with a greater pallet of soluble polymers. The nanoparticle dispersion is not expected to be affected significantly by polymer composition as nanoparticle are mixed with the polymer in flight and on the substrate. This is shown by the excellent dispersion of different nanoparticle fillers (i.e., SiO_2_, Fe_2_O_3_, TiO_2_) in PVA and PEDOT:PSS. Additional precautions and infrastructure may be required, however, when spraying harmful and/or combustible polymer solutions.

Here, TiO_2_ nanoparticles were added to the PEDOT:PSS, potentially making this an attractive nanocomposite for either a mechanically reinforced conductor [33] or a bulk heterojunction organic–inorganic solar cell [35]. By measuring the electric resistance across the diameter of these paper-bound nanocomposites (~10^4^ Ω), their average conductivity (~10 S·cm^−1^) was estimated. Optimizing the filler loading, the nanoparticle material and/or substrate topography offers feasible options to further enhance the conductivity of these nanocomposites [36].

The TiO_2_–PEDOT:PSS nanocomposites on paper demonstrate also the compatibility with shadow mask use. For (micro)electronics [26] or sensors [15], location-specific deposition on substrates is required. The well-defined edges of the individual nanocomposites suggest that bleeding of the polymer solution does not occur. The preparation of finer structures, however, would give more precise insight [37].

## 4. Conclusions

In summary, a new bottom-up fabrication technique of polymer nanocomposite films, coatings, patterns and (micro)structures is introduced. It is truly single-step since nanoparticle synthesis occurs quasi-simultaneously with polymer mixing and composite deposition on a substrate. Neither post-fabrication nor vacuum conditions are required. No agglomerating nanoparticles or voids are observed even at high filler loading. Films can be tuned in thickness from nanometers to multiple micrometers consisting of a simple two-component nanocomposite or one with multiple layers of varying composition, filler content and size. No fabrication dead-time is required between subsequent layer deposition. These benefits, in addition to the large achievable coating area, enhance industrial attractiveness. The span of devices/applications that can be realized with this technique is expanded by its compatibility with the use of untreated PDMS and standard paper as substrates.

## Supplementary Materials

The following are available online at http://www.mdpi.com/1996-1944/11/7/1177/s1, Figure S1: Nanocomposite films with low PVA solution concentration (C_p_ = 1/128 wt %), Figure S2: Pure SiO_2_ nanoparticle deposition, Figure S3: Theoretical nanocomposite film thickness and Figure S4: Filler-free polymer deposition (each figure with complementary text for description and interpretation).
