# Supplementary material for: Single-Step Fabrication of Polymer Nanocomposite Films
Source: Materials (Basel). 2018 Jul 10;11(7):1177. doi: 10.3390/ma11071177 (PMC6073108; doi:10.3390/ma11071177)
Supplement: Supplementary file 1 [file materials-11-01177-s001.pdf]

Supplementary Material

Single-Step Fabrication of Polymer Nanocomposite Films

*Christoph O. Blattmann and Sotiris E. Pratsinis**

Particle Technology Laboratory ETH Zürich, Sonneggstrasse 3, 8092 Zürich, Switzerland
*pratsinis@ethz.ch

S1. Nanocomposite films with a low PVA solution concentration (C_p =1/128 wt%)

Figure S1 shows SEM cross-section images of a SiO_2–PVA nanocomposite film prepared in a single-step (Figure 1) using a low PVA concentration (C_p = 1/128 wt %) in the sprayed polymer solution and depositing for t = 4 min. Its average thickness (~370 nm, yellow arrows in Figure S1a) is as expected slightly thinner but similar to that of films prepared for same deposition duration where polymer solutions with higher C_p (1/16 and 1/32 wt %) are used (Figure 2). The planar surface seen in Figures S1a is identical to that obtained with higher C_p (Figure 2). Regardless of the low C_p of the sprayed polymer solution, the SiO_2 nanofiller (bright spots) is homogeneously distributed within the PVA matrix (Figure S1b) also in agreement with what is obtained in films where higher C_p is used (Figure 2).

Such films are expected to exhibit a filler content that is greater than 25 vol% as this is obtained already when more PVA (C_p =1/32 wt %, Figure 2) is added. Such high filler loading with this small nanoparticle size (d_{SiO2} = 20 nm) is difficult to obtain [1] especially when homogeneity is demanded. The flexibility to add such high filler content significantly enhances the attractiveness of this already rapid and single-step fabrication.

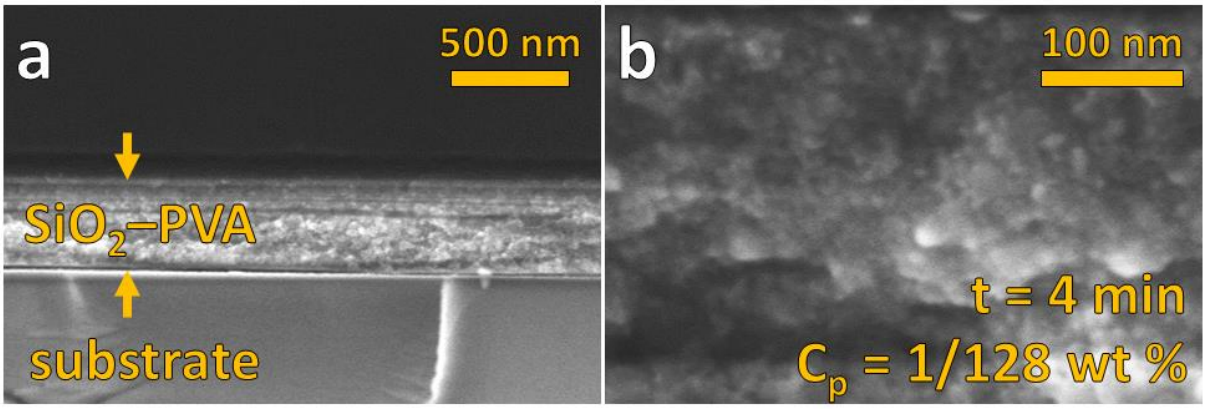

Figure S1. SEM cross-section images of a SiO_2–PVA nanocomposite film prepared with a polymer solution containing C_p = 1/128 wt % PVA and depositing for t = 4min on a glass substrate. Bright spots in **a** and **b** correspond to SiO_2 filler within the PVA matrix (light grey). Yellow arrows in a indicate the film thickness.

S2. Pure SiO_2 nanoparticle deposition

High versatility of single-step nanocomposite fabrication enables the preparation of pure nanoparticle films by spraying a polymer-free solution (C_p = 0 wt %) during nanoparticle deposition. Figure S2 shows SEM cross-section images of such a film at different magnifications prepared with SiO_2 nanoparticles (t = 4 min, C_f = 0.25 M). These films exhibit a rougher topography (Figure S2a) due to nanoparticle agglomerates protruding from its surface. Such a protrusion is enlarged in Figure S2c. There one can see the nanoparticulate features covering its surface. Their dimensions are in agreement with the expected nanoparticle size (d_{SiO2} = 20 nm).

These films exhibit a compact morphology where individual nanoparticles within the bulk film cannot be recognized. This contrasts starkly to the SiO_2–PVA nanocomposites (Figure 2 and Figure S1) where individual nanoparticles are seen throughout the entire film cross-section. The compact film morphology also differs substantially from the highly porous ones obtained during regular flame aerosol deposition [2].

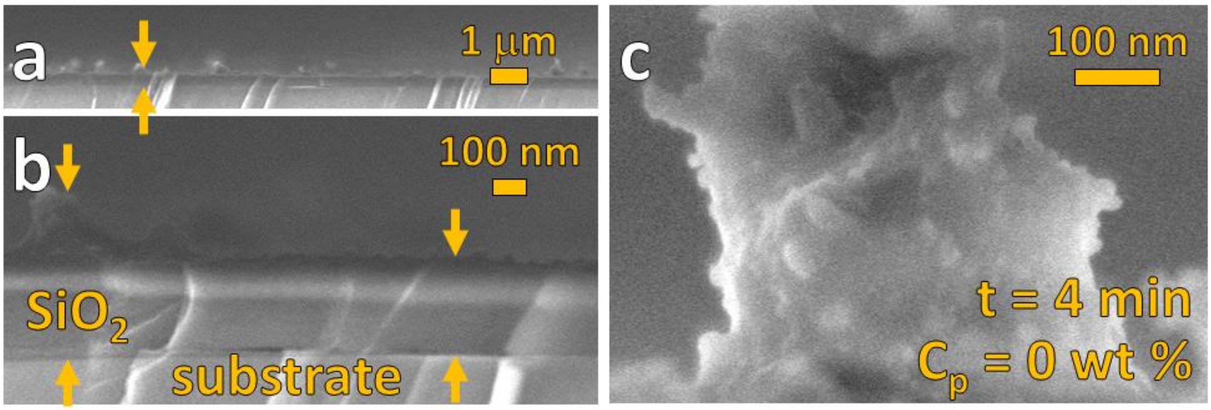

Figure S2. SEM cross-section images of a pure SiO_2 nanoparticle film prepared with a polymer-free PVA solution (C_p = 0 wt %) and depositing for t = 4 min. The topography is much rougher (**a**) due to protrusions (**c**) from its surface. The compact film morphology can be seen in **b**. The yellow arrows in **a** and **b** indicate the film thickness.

S3. Theoretical nanocomposite film thickness

Changing the filler content in SiO_2–PVA nanocomposites by *only* varying the polymer solution concentration C_p (i.e. identical flame synthesis precursor concentration C_f and deposition duration t) leads to a constant total amount of deposited/incorporated nanoparticles (i.e. m_{SiO2} = constant) but unavoidably alters the resulting film thickness (higher filler loading for lower C_p of polymer solution).

Figure S3 shows the theoretical PVA nanocomposite thickness as a function of filler content (red line) for a film exhibiting m_{SiO2} = 0.115 mg of SiO_2 and covering an area of A_{NC} = 4 cm^2. This film thickness does not scale linearly with filler content. It changes drastically for <25 vol % nanoparticles. At higher loading this change becomes increasingly smaller until a compact SiO_2 layer is obtained (i.e. 100 vol %).

Experimentally derived values for SiO_2–PVA nanocomposites (circles) prepared with C_p = 1/8 and 1/32 wt % and depositing for t = 4 min (Figure 2c,e) are in good agreement with the theoretical trend. This indicates that the amount of SiO_2 filler depositing on the substrate is independent of added PVA. As a result, once the SiO_2 deposition rate (here: ~7.2 $\mu g \cdot cm^{-1} \cdot min^{-1}$) is determined, one is able to quickly predict the filler loading solely from the nanocomposite film thickness and deposition duration t.

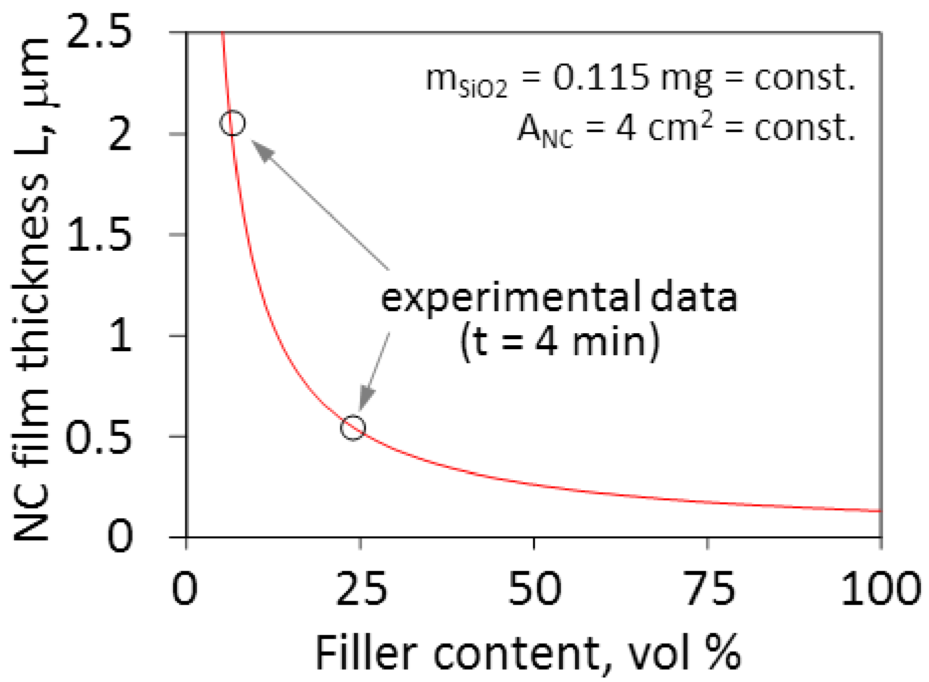

Figure S3. The non-linear correlation of theoretical film thickness (red line) as a function of filler content for PVA nanocomposite films containing identical amount of SiO_2 nanoparticles (m_{SiO2} = 0.115 mg = constant) and covering the same area (A_{NC} = 4 cm^2 = constant). The thickness changes less at high filler content agreeing well with experimental measurements (circles) shown in Figure 2.

S4. Filler-free polymer deposition

Single-step fabrication of polymer nanocomposites (Figure 1) is capable of achieving agglomerate-free films with extreme variability of filler content. It is easily tuned by (1) increasing polymer solution concentration (C_p), (2) its feed-rate or (3) the rate at which nanoparticles are synthesized. On the one extreme, filler-free polymers can be prepared by either employing a particle-free flame or eliminating the flame altogether. Figures S4 shows SEM cross-section images of pure PVA films prepared on glass substrates with a particle-free flame. The deposition duration t is identical (2 min) for all three images whereas the polymer solution concentration C_p is varied between 1/8 (Figure S4a), 1/16 (Figure S4b) and 1/32 wt % (Figure S4c).

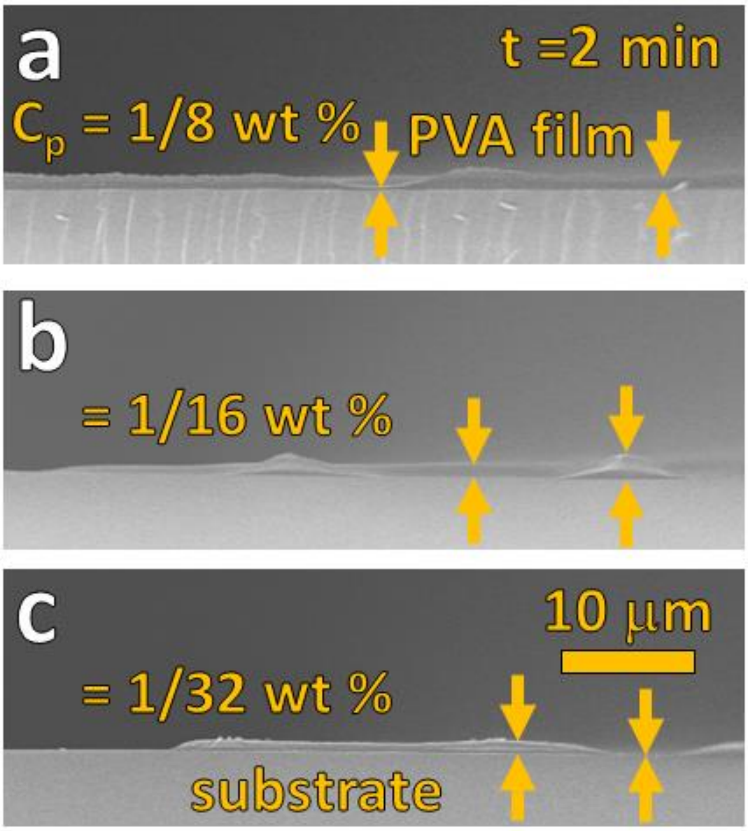

Figure S4. Filler-free PVA films prepared with a polymer solution concentration C_p =1/8 (**a**), 1/16 (**b**) and 1/32 wt % (**c**) exhibit an uneven surface topography. The yellow arrows indicate the strong variation in film thickness. The deposition duration (t = 2 min) is same for all films. The scale bar shown in **c** is identical for all images.

In contrast to nanocomposites with filler (Figure 2-4, Figures S1), these exhibit a less planar surface topography for all C_p. In fact, there even are areas where PVA does not cover the substrate. More planar film surface obtained with nanoparticle addition (Figure 2) stems from the surface wetting induced by nanoparticles [3]. Nevertheless, optimization of the fabrication by adjusting the substrate temperature or eliminating the particle-free flame may

improve the film formation for pure polymers [4]. Alternatively, the deposition can be carried out on a vibration-assisted substrate as this has shown efficacy for preparing homogeneous polymer fims with and without fillers [5].

S5. References

1. Camenzind, A.; Caseri, W.R.; Pratsinis, S.E. Flame-made nanoparticles for nanocomposites. *Nano Today* **2010**, *5*, 48-65.
2. Mädler, L.; Roessler, A.; Pratsinis, S.E.; Sahm, T.; Gurlo, A.; Barsan, N.; Weimar, U. Direct formation of highly porous gas-sensing films by in-situ thermophoretic deposition of flame-made Pt/SnO_2 nanoparticles. *Sens. Actuator. B-Chem.* **2006**, *114*, 283-295.
3. Cruz, S.; Rocha, L.A.; Viana, J.C. Enhanced printability of thermoplastic polyurethane substrates by silica particles surface interactions. *Appl. Surf. Sci.* **2016**, *360*, 198-206.
4. Felton, L.A. Mechanisms of polymeric film formation. *Int. J. Pharm.* **2013**, *457*, 423-427.
5. Soltani-Kordshuli, F.; Zabihi, F.; Eslamian, M. Graphene-doped PEDOT:PSS nanocomposite thin films fabricated by conventional and substrate vibration-assisted spray coating (svasc). *Eng. Sci. Technol. Int. J.* **2016**, *19*, 1216-1223.
